# Role of *abd-A* and *Abd-B* in Development of Abdominal Epithelia Breaks Posterior Prevalence Rule

**DOI:** 10.1371/journal.pgen.1004717

**Published:** 2014-10-23

**Authors:** Narendra Pratap Singh, Rakesh Kumar Mishra

**Affiliations:** Centre for Cellular and Molecular Biology, Council of Scientific and Industrial Research, Hyderabad, India; New York University, United States of America

## Abstract

Hox genes that determine anteroposterior body axis formation in all bilaterians are often found to have partially overlapping expression pattern. Since posterior genes dominate over anterior Hox genes in the region of co-expression, the anterior Hox genes are thought to have no function in such regions. In this study we show that two Hox genes have distinct and essential functions in the same cell. In *Drosophila*, the three Hox genes of the bithorax complex, *Ubx*, *abd-A* and *Abd-B*, show coexpression during embryonic development. Here, we show that in early pupal abdominal epithelia, *Ubx* does not coexpress with *abd-A* and *Abd-B*, while *abd-A* and *Abd-B* continue to coexpress in the same nuclei. The *abd-A* and *Abd-B* are expressed in both histoblast nest cells and larval epithelial cells of early pupal abdominal epithelia. Further functional studies demonstrate that *abd-A* is required in histoblast nest cells for their proliferation and suppression of *Ubx* to prevent first abdominal segment like features in posterior segments while in larval epithelial cells it is required for their elimination. We also observed that these functions of *abd-A* are required in its exclusive as well as the coexpression domain with that of *Abd-B*. The expression of *Abd-B* is required in histoblast nest cells for their identity while it is dispensable in the larval epithelial cells. The higher level of *Abd-B* in the seventh abdominal segment, that down-regulates *abd-A* expression, leads this segment to be absent in males or of smaller size in females. We also show that *abd-A* in histoblast nest cells positively regulates expression of *wingless* for the formation of the abdominal epithelia. Our study reveals an exception to the rule of posterior prevalence and shows that two different Hox genes have distinct functions in the same cell, which is essential for the development of abdominal epithelia.

## Author Summary

The spatially non-overlapping function of Hox genes is known to determine Antero-posterior body axis in all the bilaterians. The expression of Hox genes is found to be overlapping in several cases. According to the posterior prevalence rule, posterior Hox genes suppress the function of anterior Hox genes in the overlapping expression domains. Our findings show an exception to the rule of posterior prevalence. We show that in the overlapping expression domains of *abd-A* and *Abd-B* in early pupal abdominal epithelia, both the genes have essential roles. While *abd-A* is required for cell proliferation, *Abd-B* determines the segmental identity.

## Introduction

Anteroposterior (AP) body axis in all the bilaterians is determined by a set of homeobox (Hox) containing genes, the Hox genes [1]. The eight Hox genes in *Drosophila* are arranged in two clusters, the Antennapedia complex (ANT-C) and the bithorax complex (BX-C) [2]–[4]. BX-C has three Hox genes *Ultrabithorax* (*Ubx*), *abdominal-A* (*abd-A*) and *Abdominal-B* (*Abd-B*) [3], [5]. During development, Hox genes express in a collinear manner where the order of genes in the genomic locus is similar to their spatial expression pattern along the AP axis of the embryo [6]–[9]. This expression pattern of Hox genes determines the identity of the body segments along the AP body axis [10], [11]. In several instances, expression of Hox genes is found to be overlapping [9]. In such cases, posteriorly expressed Hox genes are known to suppress function of anterior genes at transcriptional or post-translational level of gene regulation, a phenomenon known as posterior dominance [10], [12]–[15]. Therefore, the function of the anterior Hox gene in such regions of overlapping expression with the posterior Hox gene is thought to be irrelevant. This is supported by the observation that mutants for anterior Hox genes do not show distinct phenotypes in the region of overlapping expression up to early larval stage and die later during development. Owing to this, the role of anterior Hox genes in the region where they co-express with posterior ones has not been investigated extensively during larval and pupal stages. A few studies, however, suggest that anterior Hox genes can have non-homeotic functions in the region of co-expression with posterior Hox genes [16], [17].

In order to investigate the role of three Hox genes *Ubx*, *abd-A* and *Abd-B* in their non-overlapping and overlapping domains of expression, we analyzed their expression pattern in the early pupal abdominal epithelial cells. Each abdominal segment at larval stage has two cell types the polytenized larval epithelial cells (LECs) and diploid histoblast nest cells (HNCs). The HNCs are maintained in a quiescent state during the larval development and proliferate during the early pupal development to differentiate into adult abdominal epithelial cells [18], [19]. During this process, HNCs induce apoptosis in the LECs and replace them with a layer of abdominal epithelium. For consistency we have used early pupal abdominal epithelia of 0 to 32 h after puparium formation (APF) having both LECs and HNCs. The abdominal epithelia formed after 32 h APF by proliferation and differentiation of HNCs and removal of LECs are termed as pupal abdominal epithelia. These pupal abdominal epithelial cells further develop into adult abdominal epithelia with features like bristles and pigmentation. We found that *Ubx* is expressed only in HNCs and LECs of first segment of early pupal abdominal epithelia and does not overlap with *abd-A* or *Abd-B*. On the contrary, *abd-A* co-expresses with *Abd-B* in HNCs and LECs. Here we show that in the coexpressing HNCs, *abd-A* is required for formation of adult abdominal epithelia while *Abd-B* is required for its identity. We also observed that the higher expression of *Abd-B* in abdominal segment 7 suppresses expression of *abd-A* that leads to smaller segment in females and complete elimination in males. These findings, for the first time, show that *abd-A* is required for abdominal epithelia formation not only in its exclusive expression domain but also in the region where it overlaps with *Abd-B*.

## Results

### Expression analysis of *Ubx*, *abd-A* and *Abd-B* in early pupal abdominal epithelia

**Figure 1:**
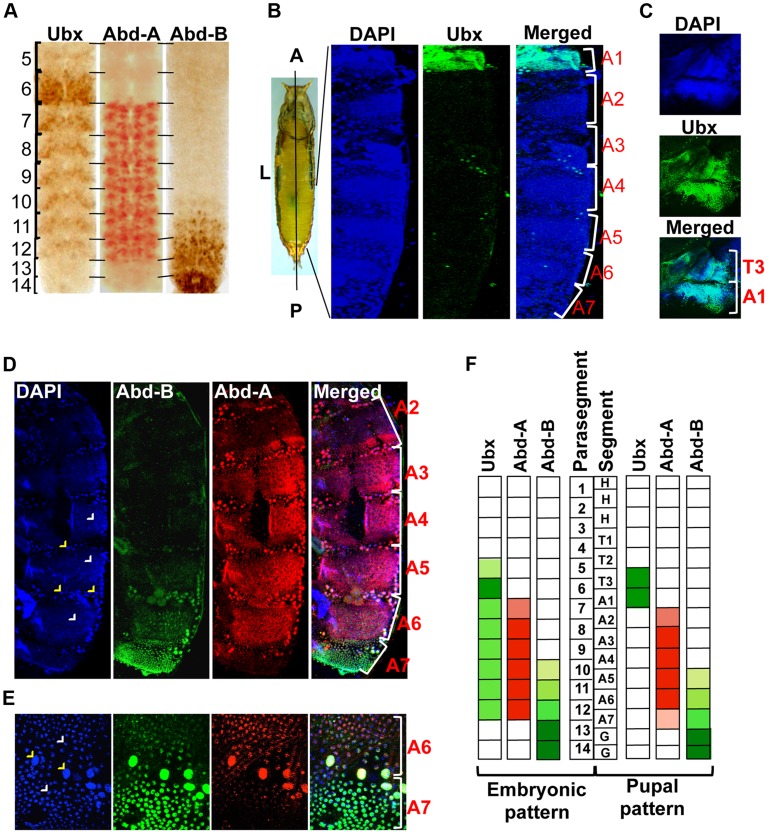
Immunostaining of Ubx, abd-A and Abd-B. A) Expression pattern of three BX-C genes are shown in parasegments (PS) of embryonic CNS, placed anterior towards up. B) Whole pupae were cut longitudinally from dorsal side to isolate two halves of early pupal abdominal epithelia. The expression pattern of Ubx is seen only in LECs and HNCs of A1 and not in posterior segments. The early pupal abdominal epithelium is placed with anterior at the top. C) The expression of Ubx is also seen in T3 apart from A1 segment. D) Expression of Abd-A is seen in LECs (yellow arrowheads) and HNCs (white arrowheads) of A2–7, although the expression in A2 and A7 is lower in comparison of other segments. Expression pattern of Abd-B shows a gradient from A5 to A7, where A5 shows minimum and A7 shows maximum expression. E) Magnified picture of A6 and A7 segment shows the expression of Abd-A and Abd-B in LECs (yellow arrowheads) and HNCs (white arrowheads) of A6 and A7. The expression of Abd-A is weaker in A7 in comparison to that of in A6. F) A pictorial representation of expression pattern of the three Hox genes in embryonic PS compared with their expression in the corresponding segments in early pupal abdominal epithelia.

Three Hox genes of the BX-C in *Drosophila melanogaster* determine the identity of third thoracic and all abdominal segments [9], [20]. The identity of third thoracic (T3) and first abdominal segment (A1) is determined by *Ubx*, A2 to A4 by *abd-A* and A5 to A9 by *Abd-B* [9]. Although these Hox genes regulate the identity of specific segments in adults, their expression is not restricted only to the corresponding parasegments (PSs) in embryos [7], [21], [22]. The *Ubx* gene is expressed from PS5 to PS12, *abd-A* expresses from PS7 to PS12 while *Abd-B* from PS10 to PS14 in embryos [6], [7], [23]. This results in the overlap of *Ubx* with *abd-A* in PS7 to PS9, and all three Hox genes in PS10 to PS12 (**Figure 1A** **, Figure S1A and B**) [8], [21]. This overlapping expression is seen not only in the same parasegment but also in the same cells (**Figure S1A and B**), raising the question of why an anterior Hox gene should express in the domain of a posterior Hox gene if it has no apparent functions there.

We further explored if the overlapping expression pattern seen during embryonic development also persists in LECs and HNCs of early pupal abdominal epithelia. In immunostaining experiments, we observed the expression of Ubx in T3 and A1 segment, Abd-A from A2–A7 and Abd-B from A5–A7 (**Figure 1B, C and D** **respectively**), which is similar to earlier observations [24], [25]. The expression of all the three genes is seen in bigger polytenised LECs (yellow arrowheads) and smaller diploid HNCs (white arrowheads). In contrast to the embryonic expression pattern, Ubx is observed only in third thoracic (T3) and first abdominal segment (A1) and not in any of the posterior segments (**Figure 1B and C**). This suggests that Ubx expression does not overlap with the other two genes while overlapping expression of Abd-A and Abd-B is seen from A5 to A7 (**Figure 1D**). A closer look reveals that the expression of *abd-A* is not uniform and shows very weak expression in A2 and A7 in comparison to other segments (**Figure 1D**). The expression of *Abd-B* continues to show a lower to higher gradient from A5 to A7 as seen in embryonic CNS (**Figure 1A and D**). Furthermore, we observed that from A5 to A7 the Abd-A and Abd-B expression coexists not only in same segment but also in the same nuclei (**Figure 1E**). These observations establish that, unlike the embryonic expression pattern, at pupal stage the expression of *Ubx* does not overlap with *abd-A* or *Abd-B* while the overlap between *abd-A* and *Abd-B* persists in LECs and HNCs of early pupal abdominal epithelia (**Figure 1F**).

### Functional analysis of *abd-A* in adult abdominal epithelia development

**Figure 2:**
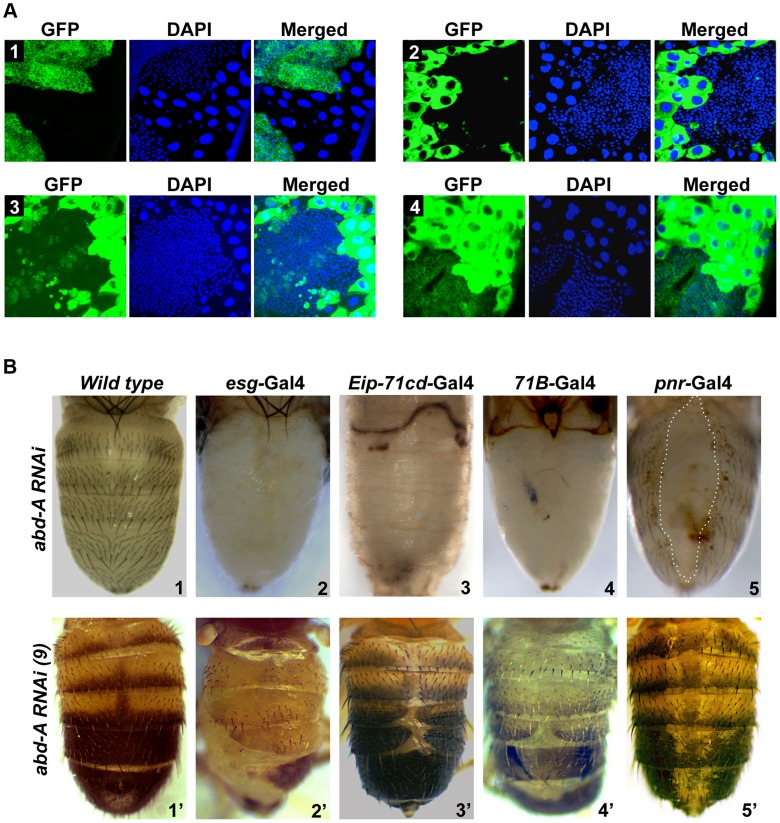
*abd-A* is required for abdominal epithelia development. A1–4) Expression of *esg*, *Eip-71CD, 71B* and *Pnr* Gal4 lines, respectively, are shown using UAS-GFP. The expression of *esg-*Gal4 is seen in HNCs of 22 h APF, *Eip-71CD*-Gal4 only in LECs and *71B* and *Pnr* Gal4s in both LECs and HNCs of 26 h APF. B1-5) Abdominal segments of wild type (B1) and *abd-A RNAi* pharates using various Gal4s are shown as indicated. B1′–5′) Phenotype of abdominal tergites in wild type and weaker *abd-A RNAi* line using various Gal4 lines, as indicated on the top of the panel. Abdominal part of pharates and adult flies are placed facing dorsal side and anterior at the top.

To assess the role of *abd-A* and *Abd-B* in both overlapping and non-overlapping expression domains, we chose to knock down *abd-A* and *Abd-B* in HNCs and LECs using UAS-*RNAi* and Gal4 approach. We used *esg-*Gal4 and *Eip*-*71CD-*Gal4 for exclusive knockdown in HNCs and LECs, respectively and *71B-*Gal4 and *Pnr-*Gal4 for knockdown in the both cell types [26], [27] (**Figure 2** **A1-4**). The *esg-*Gal4 is known to express in all histoblast nest cells in early pupa (before 24 h), which fades away later in development (**Figure 2** **A1**) [19]. We found that the expression of *71B-*Gal4 is seen mainly in larval epithelial cells till 18h APF but later it also starts expressing in HNCs at low levels and increases with time. Interestingly, its expression in HNCs is stochastic and not uniform as seen in *esg-*Gal4. (**Figure 2** **A3 and Figure S2**). The expression of *Pnr-*Gal4 is not seen in early pupa and starts expressing in LECs after 14 h APF, which later increases and is seen in all LECs and in few rows of HNCs that are leading towards dorsal midline (**Figure 2** **A4 and Figure S3**).

Knockdown of *abd-A* in HNCs using *esg-*Gal4 driver shows lethality at larval stages and only ∼20% larvae pupated although none of them hatch. These pupae showed complete loss of abdominal epithelia in the expression domain of *abd-A* (A2–A7) but not in A1 (**Figure 2** **B2**) suggesting this to be an *abd-A* specific phenotype. The loss of epithelia was observed from both dorsal and ventral sides of the segment and no tergite or sternite was seen in these segments (**Figure 2** **B2 and Figure S4**). This brings out the critical role of *abd-A* in HNCs for development of adult abdominal epithelia at pupal stage of development. The knockdown of *abd-A* in LECs using *Eip-71CD-*Gal4 shows complete lethality at very early stage (before 24 h) of pupal development. These pupae did not even grow enough to show any epithelia formation (**Figure 2** **B3**). The simultaneous knocking down of *abd-A* in both LECs and HNCs using *71B-*Gal4 also shows developmental arrest at pupal stage leading to lethality. Manually eclosed pharates show loss of abdominal epithelia similar to what we see with *esg-*Gal4 (**Figure 2** **B4**). Similarly, the *abd-A* knockdown using *Pnr-*Gal4 also showed lethality at pupal stage but manually eclosed pupae showed dorsal closure defect of abdominal epithelia (DDA) (**Figure 2** **B5**: marked by dotted line). In this case we observed loss of epithelia only close to dorsal mid line, which corresponds to the expression pattern of this Gal4 driver in LECs and HNCs.

We also generated milder *abd-A* RNAi lines (see materials and methods) and used them to knockdown *abd-A* in HNCs and LECs. As expected, these lines gave less severity and penetrance of the phenotypes. The phenotype of one of the lines is shown in figure 2 B2′-5′. Knockdown of *abd-A* in HNCs using this line with *esg-*Gal4 shows less lethality at pupal stage and adult flies show partial loss of abdominal epithelia in adults, which is always restricted to A2–6 (**Figure 2** **B2′**). The adult abdominal epithelium of the knockdown flies also show lesser and smaller bristles in comparison to the wild type flies (**Figure 2** **B2′ and 2B1′ respectively**). This indicates the homeotic transformation of the epithelia of posterior segments into A1 segment that has smaller bristles. This surprised us because in the *abd-A* knockdown the features of posterior segments transform into features similar to *Ubx* expression domain A1, although we did not observe *Ubx* expression in HNCs of these segments. We reasoned that the knockdown of *abd-A* might be leading to the derepression of *Ubx* in posterior segments thus showing this phenotype. To test this, we did immunostaining of Ubx in *abd-A* RNAi background. We observed the expression of *Ubx* only in HNCs of posterior segments (**Figure S5**), which was not seen in wild type epithelia (**Figure 1B**), suggesting that in wild type epithelia *abd-A* suppresses expression of *Ubx* in its expression domain.

Furthermore, knockdown of *abd-A* using *Eip-71CD-*Gal4 and *71B-*Gal4 with milder *abd-A* RNAi line also showed viability and DDA phenotypes in adults (**Figure 2** **B3′–4′**). The DDA phenotype is always restricted to A2 to A6 segment. Knockdown of *abd-A* by *Pnr*-Gal4 shows loss of pigmentation across the dorsal midline, suggesting that the expression of *abd-A* is also required for pigmentation of adult cuticle (**Figure 2B** **5′**). The phenotype of these *abd-A RNAi* experiments shows that the knockdown of *abd-A* in HNCs leads to a loss of epithelial cells while that in LECs causes DDA phenotype. This indicates that the level of *abd-A* is critical in HNCs and LECs during development of adult abdominal epithelia at pupal stage. Interestingly this role of *abd-A* in epithelia formation is not limited only in its exclusive expression domain but also in the domain where it overlaps with *Abd-B* (A5 and A6 segments).

### Role of *abd-A* expression in the HNCs

**Figure 3:**
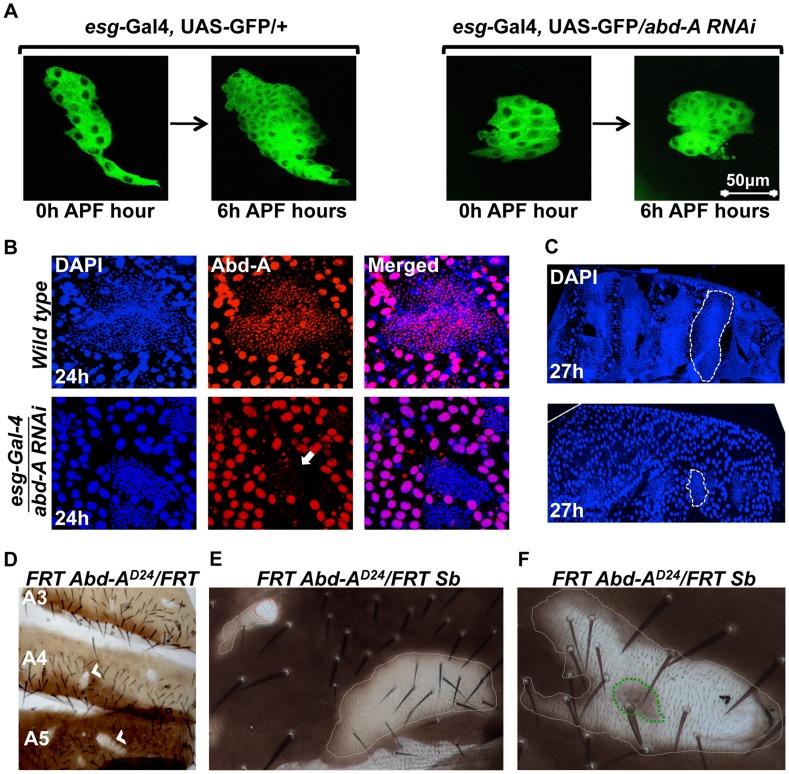
*abd-A* is required in HNCs for their proliferation. A) Live cell imaging using *esg-*Gal4 UAS*-*GFP shows reduced proliferation of HNCs in the case of *abd-A RNAi*. B) Immunostaining of 24 h old pupal abdominal epithelial cells in wild type and in the *abd-A RNAi* with *esg-*Gal4 show cell type specific loss of *abd-A* and less number of HNCs in the case of *abd-A RNAi* (white arrow) in comparison to wild type. The DAPI stained nuclei of LECs (bigger in size) in wild type epithelia are not spherical and show irregular shape indicating HNC induced cell death while in the case of *abd-A RNAi* these nuclei show round shape. C) Lower panel of 27 h APF abdominal epithelia (placed anterior left) in *abd-A RNAi* using *esg*-Gal4 show less number of HNCs (marked by dotted lines) and more number of LECs in comparison to wild type (upper panel). D) Mitotic clones of *abd-A* mutant are seen in all three abdominal segments. E) The *abd-A* clones white in color are small without bristles (red dotted lines) while mitotic clones with lighter pigmentation show small bristles (white dotted lines). F) This clone of *abd-A^D24^* is made using stubble (*Sb*) mutation (gives stubble bristle) on other homologous chromosome. We see a stubble bristle in middle of the clone while normal bristles are mainly present in the light pigmented areas and white patch show rare or no bristles.

We further assessed the role of *abd-A* in HNCs using live cell imaging. The HNCs are quiescent during larval stage and get signal to proliferate as soon as pupation starts. The proliferation of HNCs is biphasic, where in the first phase (4–12 hours APF), cells divide without growing much in size while in the second phase (15–36 hours APF) cells divide normally and make complete epithelia [19]. In the *abd-A* knockdown using *esg-*Gal4 the live cell imaging of HNCs showed almost 50% reduction in the proliferation of HNCs during first six hours of the first phase of proliferation (**Figure 3A**). There are 16 HNCs at 0 h APF, after 6 h of proliferation their number become 60±5 HNCs in the case of *esg-*Gal4 while it is only 31±5 in the case of *abd-A* RNAi. We also observed that about 30% of the analyzed HNCs show complete arrest at this stage and do not proliferate. Further analysis of LECs and HNCs in these pupae also showed that non-proliferating HNCs are unable to eliminate LECs, which results in the arrest in development of abdominal epithelia (**Figure 3B and C**). This clearly shows that *abd-A* is required in HNCs for their proliferation during early pupal development.

To further analyze the role of *abd-A* in the HNCs during the development of adult epithelia, we made mitotic clones of *abd-A* using its loss of function allele, *abd-A^D24^* [28]. Mitotic clones were observed in all segments from A2 to A6 implying a common role of *abd-A* in all these segments (**Figure 3D**). Mitotic clones are seen as either small white patches on the tergites without bristles (**Figure 3E** **, red dotted line**) or relatively larger light pigmented regions with small bristles (**Figure 3E** **, white dotted line**). One of such white clones shows bristles only at the places where pigmentation is low while white areas are devoid of bristles (**Figure 3F**). In the middle of the clone we also see a pigmented patch (marked with green dotted line) with stubble bristle representing an *abd-A* positive region. The small bristles in the mitotic clones suggest that these cells are transformed into A1 like cells as seen on knocking down of *abd-A* in HNCs (**Figure 2B** **2′**). We further extended our analysis and gave heat shock at 0 h, 12 h, 24 h and 36 h APF to evaluate the role of *abd-A* in HNCs during pupal development. We observed that mitotic clones show phenotypes only in the flies that were given heat shock at 0 h, 12 h APF and not later stages. All pupae (40 out of 40) that were given heat shock at 0 h APF show phenotype in mitotic clones of adult flies while only 15% of (6 out of 40) pupae that were given heat shock at 12 h APF show phenotype in mitotic clones. These results confirmed the *abd-A* RNAi result that *abd-A* is required in HNCs for identity and proliferation during the first phase of HNC proliferation. These observations also suggest that expression of *abd-A* is required for normal size bristles not only in its exclusive expression domain but also in the expression domains (A5 and A6) where it overlaps with *Abd-B*.

### Role of *abd-A* expression in the LECs

**Figure 4:**
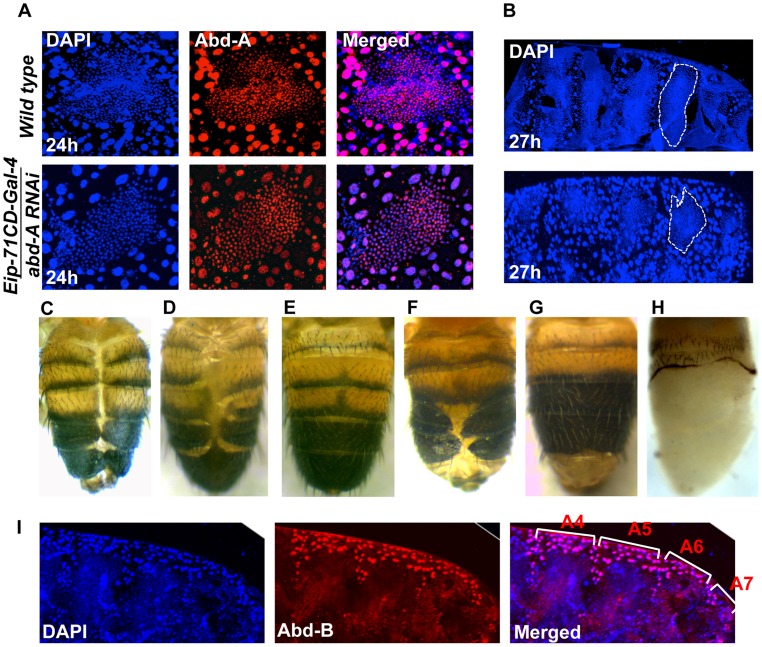
Expression of *abd-A* is required in LECs for their removal. A) *abd-A RNAi* using *Eip-71CD-*Gal4 show LECs specific reduction of Abd-A level (lower panel) in comparison to wild type (upper panel). Loss of *abd-A* from LECs shows spherical morphology of nuclei suggesting that the cell death is not induced in these cells. B) The *abd-A* RNAi (lower panel) in LECs show more number of LEC nuclei and less number of HNC nuclei as compared to the wild type 27 h APF epithelia. C) Over expression of antiapoptotic protein P35 in LECs using *Eip-71CD-*Gal4 show DDA phenotype. D) Knockdown of *abd-A* in LECs by over expressing miR-iab-8-5p using *Eip-71CD-*Gal4 shows DDA phenotype while over expression of miR-iab-4-5p does not show DDA phenotype (E). F) A cis-regulatory mutant *Mcp^H27^ Fab-7^1^* also shows segment specific DDA phenotype. G) We observed that the DDA phenotype of *Mcp^H27^ Fab-7^1^* is rescued on knocking down *Abd-B* using *Eip-71CD-*Gal4. H) Ectopic expression of *Abd-B* in LECs using *Eip-71CD-*Gal4 shows complete loss of abdominal epithelia from A2 to A7 segment of pharate. I) Immunostaining of abdominal epithelia at 26 h (APF) shows higher expression of Abd-B (red) in LECs (bigger nuclei-stained with DAPI-blue in color) from A4 to A6 in comparison to wild type (Figure 1C). The early pupal abdominal epithelium is placed posterior at right and lateral on the top.

We investigated the role of *abd-A* in LECs by knocking down *abd-A* using *Eip-71CD-* Gal4. Since the original strong *abd-A* RNAi line gave early pupal lethality, we used a milder RNAi line. In this case also, we observed LEC specific reduction in *abd-A* expression (**Figure 4A**). These LEC nuclei do not show the distinct apoptotic feature of irregular nuclear morphology, unlike what is seen in wild type nuclei (**Figure 4A**). The development of these pupae was arrested during early pupal stage and we see that HNCs do not grow and LECs are not removed as compared to wild type (**Figure 4A and B**). This suggests that the loss of *abd-A* prevents cell death in LECs and, thereby, leaves no space for HNCs to proliferate and make complete abdominal epithelia. To confirm if the persistence of LECs during early pupal development leads to DDA phenotype as seen in *abd-A* knockdown we over-expressed anti-apoptotic factor P35 in LECs using *Eip-71CD* Gal4. These flies show DDA phenotype in all the abdominal segments (**Figure 4C**) similar to what is seen in *abd-A RNAi* using *Eip-71CD-* Gal4 (**Figure 2B** **3′**). This demonstrates that inefficient clearing of LECs prevents HNCs from growing and leads to fusion of abdominal epithelia at dorsal midline resulting in DDA phenotype.

Finally, to validate the *abd-A RNAi* results we also carried out loss of *abd-A* function by other independent ways. As miR-iab-8-5p is known to knock down *Antp*, *Ubx* and *abd-*A [29], [30], we overexpressed this miRNA using *Eip-71CD-*Gal4. Here we observed pupal lethality with few flies emerging with DDA phenotype, similar to what is seen in corresponding *abd-A* RNAi flies (**Figure 4D** **and** **2** **B3′, respectively**). In the control experiment with miR-iab-4-5p over expression, which knocks down only *Antp* and *Ubx* but not *abd-A*, this phenotype is not observed indicating that DDA phenotype seen in miR-iab-8-5p overexpression is specifically due to *abd-A* knockdown (**Figure 4E**). Similarly, we also observed the DDA phenotype in a cis-regulatory mutant, *Mcp^H27^ Fab7^1^*, with 100% penetrance (**Figure 4F**). This mutant has deletion of boundary and PRE combination from *Mcp* and *Fab7* regions, which regulates *Abd-B* gene in segment specific manner [31], [32]. We hypothesize that deletion of these cis-regulatory elements leads to ectopic expression of *Abd-B* in LECs of anterior segment causing suppression of the anterior gene *abd-A* that results into DDA phenotype. Immunostaining for Abd-B protein in *Mcp^H27^ Fab-7^1^* mutant indeed shows ectopic and enhanced expression of *Abd-B* in LECs of A4 and A5 segments (**Figure 4I**). Further, we knocked down *Abd-B* using *Eip-71CD*-Gal4 and observed complete rescue of the DDA phenotype in almost 55% of the flies, while rest of them showed partial rescue confirming that the ectopic expression of *Abd-B* causes DDA phenotype (**Figure 4G**). To further establish *Abd-B* dependent loss of abdominal epithelia, we over expressed *Abd-B* in LECs using *Eip-71CD*-Gal4 and observed 100% lethality at pupal stage. Most of the pupae died at early stages but few (15%) of them survived till later stages and showed the anticipated loss of abdominal epithelia (**Figure 4H**). This partial to complete loss of abdominal epithelia by *Abd-B* over expression is similar to what we observed in the case of *abd-A RNAi* (**Figure 2** **B2 and 4**). These results confirm our *abd-A* knockdown results and establish that *abd-A* is required in LECs for their removal during abdominal epithelia development. Taken together, these observations suggest that *abd-A* plays dual role during development of abdominal epithelia, on the one hand it is required for proliferation of HNCs and on the other hand it is required for removal of LECs.

### Functional analysis of *Abd-B* in abdominal epithelia development

**Figure 5:**
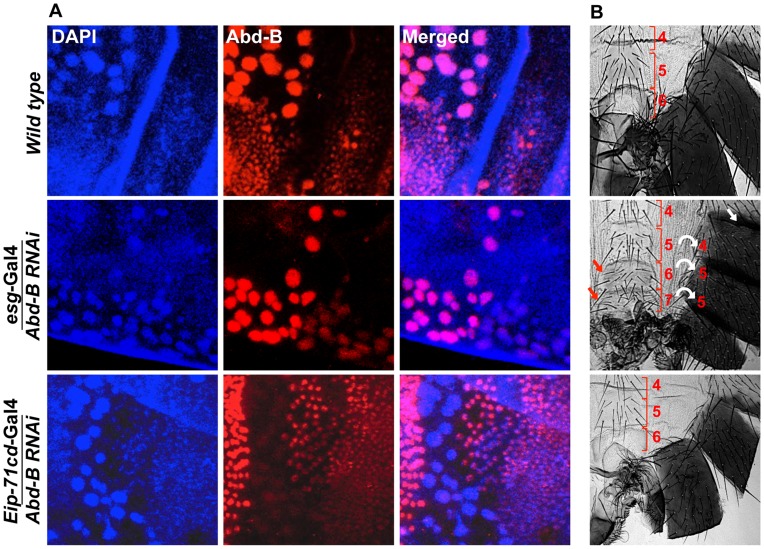
Expression of Abd-B is required in HNCs for the identity of the adult abdominal epithelia. A) *Abd-B* expresses in both cell type HNCs and LECs in wild type (top panel). Knockdown of *Abd-B* using *esg*-Gal4 show HNCs specific loss of Abd-B protein (middle panel) while in the case of *Eip-71CD-*Gal4 we see LECs specific loss of Abd-B protein (lower panel). B) It shows cuticle preparations of the adult flies of the corresponding genotype in section A. HNC specific knockdown of *Abd-B* using *esg*-Gal4 shows homeotic transformation of A5, A6 and A7 into anterior segments. The A5 segment shows loss of pigmentation (white arrow), A6 segment shows sternite bristles and we see an extra A7 with sternite bristles (red arrow). Bracket sign marks the upper and lower limits of each segment. Knockdown of *Abd-B* in LECs using *Eip-71CD*-Gal4 does not show any phenotype (lower most panel). All the cuticles in this figure and subsequent figures are placed anterior at top and dorsal at right.

We also did cell type specific knockdown of *Abd-B* to understand its role in abdominal epithelia formation. To analyze the cell type specific knockdown of *Abd-B* in HNCs and LECs we did immunostaining against Abd-B protein in early pupal abdominal epithelia in wild type and knockdown background. We observed HNC specific loss of Abd-B in the case of knockdown using *esg*-Gal4 and LEC specific loss of Abd-B with *Eip-71CD-*Gal4 (**Figure 5A**). Knockdown of *Abd-B* in HNCs of all the abdominal segments using *esg-*Gal4 driver leads to anteriorization of A5-A7 segments (**Figure 5B**) while adult epithelia formation is unaffected. The loss of pigmentation in A5 indicates its transformation to A4 while appearance of bristles in sternites of A6 is indicative of A6 to A5 transformation. We also see the homeotic transformation of A7 into A5 that is evident from its appearance as a discrete segment, which is otherwise absent in males [33], [34], and bristles in the sternite (**Figure 5B**). The transformation of these posterior segments into anterior is a typical *Abd-B* loss of function phenotype. We also observed defective genital and anal organs in these knockdown flies, which is in line with the known role of *Abd-B* in genital and anal development [35]. On the other hand, knocking down of *Abd-B* in LECs using *Eip-71CD-*Gal4 did not show any detectable phenotype (**Figure 5B**). Knock down of *Abd-B* in both HNCs and LECs driven by *71B-*Gal4 and *Pnr-*Gal4, leads to anteriorization phenotypes in A5, A6 and A7 segments (**Figure S6B and C**). The *Abd-B* knockdown using *71B-*Gal4 shows anteriorization phenotypes similar to *esg*-Gal4, however, it is milder than the *esg-*Gal4 (**Figure S6B**). This can be attributed to the lower, stochastic and late expression of *71B*-Gal4 as compared to *esg-*Gal4 (**Figure 2A** **1 and 3 and Figure S2**). The *Abd-B* RNAi using *Pnr-*Gal4 shows complete loss of pigmentation on both sides of the dorsal midline in A5 to A6 segments indicating transformation of these cells into A4 like identity (**Figure S6C**). This phenotype is seen only in cells close to dorsal mid line, which very well corresponds to expression pattern of *Pnr*-Gal4 in the HNCs (**Figure S3**). These homeotic phenotypes seen in the case of *Abd-B* knockdown by *esg, 71B* and *Pnr* Gal4 drivers establish that expression of *Abd-B* in HNCs determines the identity of abdominal epithelia. The knockdown of *Abd-B* in LECs by using *Eip-71CD-*Gal4 does not give any phenotype (**Figure 5B**) indicating that expression of *Abd-B* in these cells is dispensable. From the results of *abd-A* and *Abd-B RNAi*, we conclude that the expression of *abd-A* is critical in both HNCs and LECs for development of the complete epithelia while expression of *Abd-B* is required in HNCs for their identity but dispensable in LECs.

### *abd-A* activates *wingless* expression for development of abdominal epithelia

**Figure 6:**
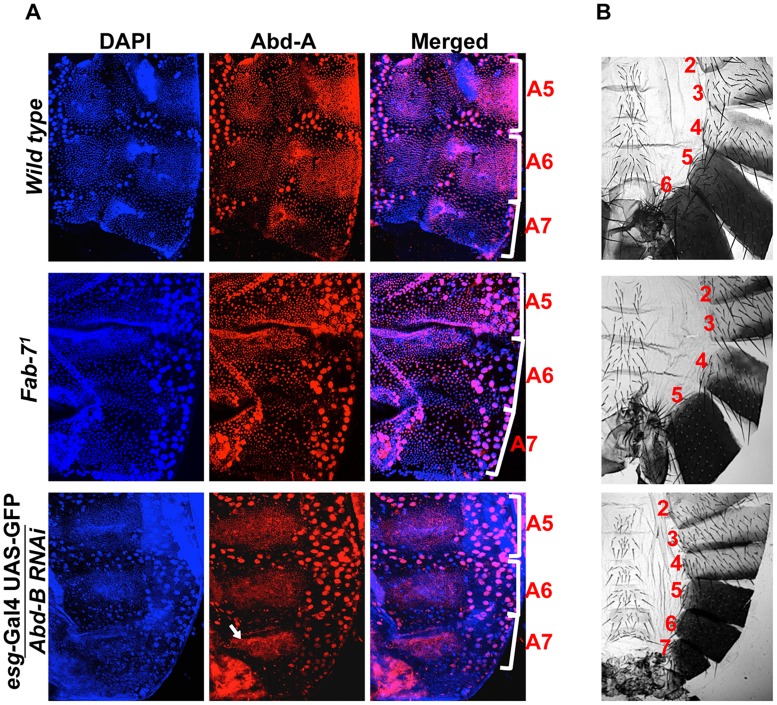
Higher expression of *Abd-B* suppresses *abd-A* expression in A7. A) Wild type abdominal epithelia show very low expression of Abd-A in A7 in comparison to A5 and A6. The *Fab-7^1^* mutant show loss of *abd-A* in both A6 and A7 segment while in the case of *Abd-B RNAi* using *esg-*Gal4 we observed derepression of *Abd-A* expression only in HNCs of A7 segment (arrow). B) Cuticle preparations of adult flies show loss of A6 segment in *Fab-7^1^* mutant (middle panel) and homeotic transformation of A5, A6 and A7 into anterior segments on *Abd-B* RNAi with *esg-*Gal4.

**Figure 7:**
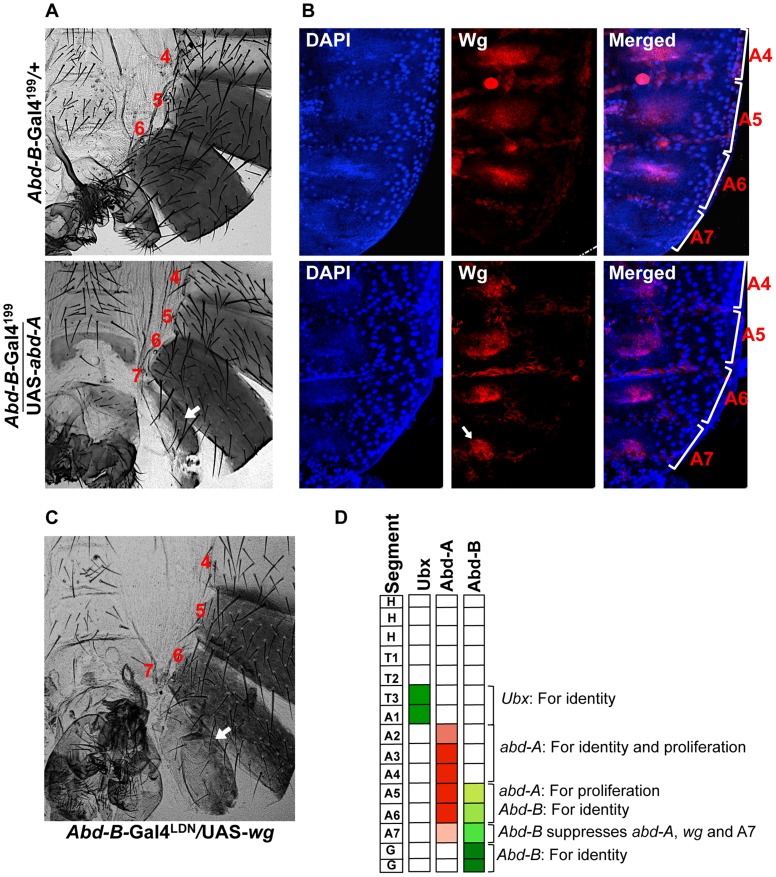
Abd-A activates *wingless* for development of abdominal epithelia. A) Over expression of *abd-A* using *Abd-B*-Gal4^199^ shows an extra segment in males (arrow in the lower panel) suggesting that *abd-A* expression is enough for abdominal epithelia formation. B) Over expression of *abd-A* in A7 using *Abd-B*-Gal4^199^ ectopically activates *wg* expression in A7 segment (arrow in lower panel). The genotypes are mentioned on left side of the A panel. C) Over expression of *wg* by *Abd-B*-Gal4*^LDN^* in A7 segment shows an extra segment. D) Summary of the hox gene expression and their role in abdominal epithelia development at pupal stage. *Ubx* expresses only in T3 and A1, the expression of *abd-A* in HNCs from A2 to A4 segment is required for both identity and proliferation while in A5 and A6 it is required for only proliferation. The expression of *Abd-B* in HNCs of A5 and A6 determines their identity while in A7 higher expression of *Abd-B* causes reduced size of this segment by suppressing the wingless pathway through suppression of *abd-A*.

Recent studies show that higher level of *Abd-B* expression in A7 segment leads to loss of HNCs and LECs that results to elimination or smaller size of A7 segment in males and females, respectively [33], [34]. While analyzing expression of *abd-A* and *Abd-B* in abdominal epithelia, we observed that in the A7 segment expression of *Abd-B* is maximum and *abd-A* expression is very weak as compared to anterior segments. (**Figure 1D**). Thus we hypothesized that *Abd-B* being a posterior gene may be suppressing *abd-A* in A7 leading to the removal of A7 segment from the abdomen of males. To understand the *Abd-B* mediated suppression of *abd-A*, we analyzed the expression pattern of Abd-A in *Fab-7^1^* mutant and *Abd-B RNAi* driven by *esg*-Gal4. The *Fab-7^1^* mutant has a deletion of boundary element which leads to higher expression of *Abd-B* in A6 segment resulting in A6 to A7 transformation and thus loss of both the segments [36]. In wild type fly *abd-A* expresses weakly in A7 as compared to A5 and A6 (**Figure 6A** **top panel**), while in *Fab-7^1^* mutant we observed very weak expression of *abd-A* in both A6 and A7, suggesting that higher levels of *Abd-B* in A6 suppresses expression of *abd-A* in this segment (**Figure 6A** **middle panel**). To further confirm this observation, we knocked down *Abd-B* in HNCs of A7 using *esg-*Gal4 and observed increased expression of *abd-A* in this segment (**Figure 6A** **lower most panel**). The gain of *abd-A* expression in A7 is specific to HNCs and it was not seen in LECs, confirming *Abd-B* dependent suppression of *abd-A*. This establishes that expression of *Abd-B* similar to that in A7 segment, suppresses *abd-A* expression while lower expression in A5 and A6 does not effect *abd-A* expression. These experiments also clearly show that loss of *abd-A* expression correlates with the loss of abdominal segment and gain of *abd-A* correlates with the gain of abdominal segment in adults (**Figure 6B**). To test if *abd-A* is required and is enough for abdominal segment formation, we overexpressed *abd-A* in A7 segment using *Abd-B*-Gal4 and observed formation of an extra segment in males (**Figure 7A** **lower panel**) and a bigger A7 segment in females with 100% penetrance (**Figure S7**). This establishes that *abd-A* expression is required and is enough for adult abdominal epithelia formation during pupal development. And in A7, higher expression of *Abd-B* suppresses *abd-A* that leads to the loss of this segment in males and smaller segment in females.

We further extended our study to understand how *abd-A* expression helps development of abdominal epithelia. Earlier studies have shown the role of Wingless (Wg) morphogen in regulating development of abdominal epithelia [37]–[39]. In A7 of male the expression of *wg* is known to be suppressed by higher *Abd-B* expression [34]. In this study we also observed that higher Abd-B levels suppress *abd-A* expression in HNCs of male A7 to promote elimination of this segment. This suggests that higher expression of *Abd-B* suppress both *abd-A* and *wg* expression in A7 for its elimination from male abdomen. We further wanted to understand if suppression of *wg* expression by *Abd-B* in A7 is through *abd-A* or independent of it. To study this, we analyzed the expression of *wg* in male A7 in the background of *abd-A* overexpression using *Abd-B* Gal4. Consistent with earlier expression we do not detect Wg morphogen in HNCs of male A7 (**Figure 7B** **upper panel**). The ectopic expression of *abd-A* leads to ectopic expression of *wg* in male A7 HNCs, clearly indicating that *wg* expression is positively regulated by *abd-A* (**Figure 7B** **lower panel**). This shows that *wg* expression in abdominal epithelial cells is activated by *abd-A* expression. To further prove that *wg* pathway is required for the formation of extra A7 segment we also did over expression of *wg* in A7 segment using *Abd-B* Gal4. We observed an extra A7 (**Figure 7C**) in all flies expressing *wg* under *Abd-B*-Gal4 similar to what we saw in over-expression of *abd-A*. This establishes that Abd-A protein activates *wg* expression for formation of abdominal epithelia. The higher expression of *Abd-B* in A7 of male induces elimination this segment by suppressing *wg* expression through suppression of *abd-A* expression (**Figure 7D**).

## Discussion

The expression pattern of anterior Hox genes is often found to be overlapping with the posterior genes although the functional importance of such an expression pattern is unknown. In this study we show that the overlapping expression of Hox genes *Ubx* and *abd-A* during embryonic development of *Drosophila* becomes spatially non-overlapping in abdominal epithelia of pupa. The expression of *Ubx* is seen in T3 and A1 and not in posterior segments while the expression of *abd-A* is seen from A2 to A7, which overlaps with that of Abd-B from A5 to A7. Using UAS/GAL4 based RNAi and FRT/FLP based genetic mosaic techniques we show that *abd-A* is required in HNCs of A2 to A6 segment for their proliferation and suppression of *Ubx* expression to provide bigger size of the bristles. This suggests that *abd-A* is required in HNCs of A2 to A6 segments for development of adult epithelia with bigger bristle size. In contrast to this, in LECs *abd-A* expression is required for apoptosis, which allow proliferation of HNCs. Here, the interesting point is that these roles of *abd-A* in abdominal epithelia development are not limited only in the segments of exclusive expression (A2 to A4) but also in the segments where its expression overlaps with that of *Abd-B* (A5 to A6).

We further show that *Abd-B* expression is seen in both HNCs and LECs of A5 to A7 and functional studies suggested that it determines the identity of HNCs but it seems to be dispensable in the LECs. Knockdown of *Abd-B* in the HNCs of A5 to A7 segment of male leads to loss of pigmentation in A5 segment, bristles in sternite of A6 segment and formation of an extra A7 with bristles. This means that loss of *Abd-B* in HNCs of A5 leads to its transformation into A4 like features, transformation of A6 and A7 into A5. Loss of function results of *abd-A* and *Abd-B* RNAi bring out the function of both the genes in the HNCs of A5 and A6 during the development of adult abdominal epithelia. The two genes not only function together in same segment but also in same nuclei for their distinct roles. This is in contrast to the posterior dominance rule where an anterior gene does not function in the presence of a posterior Hox gene.

Interestingly, the situation in A7 is just the opposite, where a higher level of *Abd-B* suppresses *abd-A* expression. This suggests that *abd-A* can coexpress with *Abd-B* in the regions where *Abd-B* expression is less than that of A7. The over expression of *abd-A* in A7 shows an additional abdominal segment in males and bigger A7 segment in female, thus finally proving that Abd-A protein is required and sufficient for adult abdominal epithelia formation. This also implies that the down-regulation of *abd-A* in A7 by Abd-B protein is required only for the reduction in the size of abdominal segment. Earlier studies have shown additional male specific roles of Abd-B protein and sex-determination regulator Doublesex that regulate the elimination of A7 in males [33], [34]. The suppression of *abd-A* in A7 is seen as loss of Abd-A protein suggesting that posterior prevalence may be operating at transcriptional or post transcriptional level and not at post-translational level where concentration dependent interaction with common cofactor(s) was implicated in deciding the dominant function of posterior Hox gene [15]. Our study, with other published data, suggests that the expression of an anterior Hox gene in the domain of posterior Hox gene is not futile and that it has adopted new roles to play in such regions [16], [17].

This study and earlier work, taken together, explain how Hox genes of the bithorax complex control development of A1 to A7 segments of adult abdominal epithelia. The expression of *Ubx*, *abd-A* and *Abd-B* is required for the identity of A1, A2–A4 and A5–A7, respectively (**Figure 7D**). We show that the expression of *abd-A* is also required in A2 to A6 for the proliferation of HNCs and elimination of LECs. In A7, however, higher levels of *Abd-B* suppress epithelia formation by down regulating expression of *abd-A*. Interestingly, smaller size of A2 compared to A3 or A4 also correlates with the relatively lower level of expression of *abd-A* in A2 as compared to that in A3 or A4. This raises the possibility of the level of Abd-A determining the sizes of segments in adult fly. While the role of Hox genes in determining the identity of body segments is well established, our findings bring into light the collective role of Hox genes in determining the size, shape and identity of body segments. Our observations are in agreement with a recent study which shows that in embryonic CNS of *Drosophila* the expression of *abd-A* is not suppressed by *Abd-B* and that the two genes coexpress in same nuclei [40]. These observations suggest that posterior dominance rule operating between *abd-A* and *Abd-B* is tissues specific and not a universal phenomenon. Further studies will be required to understand the likely functional significance of such coexpression patterns of Hox genes in other tissues and animals as well. Much of what we understand about function of Hox genes is in the context of positional identity along the AP body axis. Our study suggests the need to explore the Hox code to understand development of organs where rules like posterior prevalence may not hold.

## Materials and Methods

### Fly stocks and culture

Flies were grown in standard cornmeal yeast extract medium at 25°C unless otherwise specified. We used following stocks during this study: CantonS (*CS*) as wild type strain, *esg-*Gal4 UAS*-*GFP (Nobert Perrimon), *Abd-B-RNAi* (Vienna Drosophila RNAi Centre), *abd-A-RNAi*, UAS*-abd-A*, UAS*-Abd-B* (Yacine Graba), *Mcp^H27^ Fab-7^1^*, *Fab-7^1^*, *abd-A^D24^*, *Pnr-*Gal4 (Francois Karch), UAS*-miR-iab8-5p* (Eric Lai) and *Abd-B*-Gal4^LDN^, *Abd-B*-Gal4^199^ (Ernesto Sánchez-Herrero), UAS-*wg* and UAS-*Abd-B* (LS Sashidhara) and *w hsflp122;FRT^82^ abd-A^D24^/TM6* and *w y hsflp122;FRT^82^* GFP*/TM3, neoFRT82B Sb/TM6, 71B* Gal4, *Eip-71CD-*Gal4 were procured from the Bloomington Drosophila Stock Centre.

### Knockdown of Hox genes using UAS/Gal4 system

During this study we observed that *abd-A RNAi* line has multiple insertion of the P-element based RNAi vector in same chromosome. This gave us a clue that separating these P-elements would give us lines with less number of insertions and thus weaker *abd-A RNAi* effect. We recombined the *abd-A RNAi* carrying chromosome with the wild type chromosome and recovered lighter eye color line. In the case of *Abd-B* RNAi in *Mcp^H27^ Fab-7^1^* mutant background we recombined *Mcp^H27^ Fab-7^1^* mutant chromosome with third chromosome *Abd-B* RNAi to see the rescue in homozygous condition. In all RNAi and over expression experiments, we always used females from Gal4 lines while male from RNAi or over expression transgenic lines to employ maternal effect. All the phenotypic quantifications in the knockdown or over expression experiments is done using at least 40 larvae, pharates or adult flies of correct genotype. The pupae and larvae of correct genotypes were identified either by loss of *Tubby* marker present on balancer chromosome or Gal4 driven GFP expression.

### Immunostaining of embryos and early pupal abdominal epithelia

10–14 hours old embryos of desired genotypes were used for immunostaining as per the published protocol [7]. Monoclonal antibodies; Anti Ubx (1∶50 dilution) and anti Abd-B (1∶10 dilution) were procured from Developmental Studies Hybridoma Bank, while polyclonal goat anti *Abd-A* (1∶200) antibody from Santa Cruz (27063). The secondary antibody tagged with HRP or fluorophore was used at 1∶200 dilutions. In the case of HRP chromogenic reaction was performed to stain the embryo and then CNS of the correct stage embryos were dissected out using fine needles and images were taken by using Zeiss AxioScop 2. For imaging of embryos stained with fluorophore conjugated secondary antibody we used confocal microscope.

For immunostaining of abdominal epithelial cells during early pupal development we followed the protocol described by Wang et al. [41]. Briefly, newly pupated pupae were collected on the basis of light color and staged as per the requirement. They were cut longitudinally into two halves from dorsal side with a sharp razor. Internal organs of cut pupae were removed by flushing 1XPBS using 20 µl pipette. Cleaned abdominal epithelia were fixed in 1XPBS +4% paraformaldehyde +0.2% dioxycholic acid for one hour. Tissue was further blocked in PBSB (1XPBS +1.0% BSA) for 3 hours and then incubated overnight with the antibody in same solution. The primary antibody anti-Ubx was used at 1∶20 dilution, anti-Abd-A at 1∶100 and anti-Abd-B at 1∶10 dilution. Further tissue was washed with PBSB and subjected to secondary antibody (1∶200) for 2 to 3 hours. After this the tissue was washed and mounted in anti-fade medium with DAPI (Vectashield). Images were taken by using confocal microscope.

### Imaging of the pharates and adult abdomen

Pupae of correct stage and genotype were collected in microfuge tubes. They were washed with PBS to remove any adherent dirt and then fixed by boiling for 5 minutes in 1XPBS. The pupal case was removed gently with fine needles while in PBS to hatch the pharates [42]. The imaging of abdominal region of the fly was done by collecting the flies of interest and taking the pictures immediately or storing them at −30°C for later use. Wings and legs were cut if required for proper positioning and visualization of the abdomen. During acquisition of images light was given only from one side to avoid glittering of cuticle.

### Live cell imaging

For live cell Imaging of HNCs we followed the protocol described by Nivon et al. [19]. Newly pupated milky white color pupae of correct genotype were collected and placed in moist chamber to avoid drying. Then pupae were aligned in correct orientation so that HNCs face towards the bottom of chamber with the help of halocarbon oil. Imaging of HNCs was done in gap of two hours using Zeiss multiphoton confocal. For calculation of the change in proliferation rate we did live cell imaging of 15 *abd-A* RNAi HNCs and 10 wild type HNCs. In this analysis we did not include HNCs that showed complete arrest in the proliferation.

### Generation of mitotic clones

For making mitotic clones of *abd-A^D24^* mutant, the mutant was recombined with *FRT^82^* chromosome and recombinants were selected on G418 media. The *hsflp; FRT^82^ abdA^D24^/TM6* females were crossed with *FRT^82^*/*TM3* and *FRT^82^ Sb/TM6* males separately. Progenies were given heat shock (37°C for 2 hours) at different stages of development for activation of the flipase. We at least screened 30 flies of correct genotype of each stage to score the percent of flies showing mitotic clones.

### Preparation of fly cuticle

For making flat preparation of adult abdomen cuticle, flies of interest were collected and stored in 3∶1; ethanol:glycerol solution until use. Flies were heated at 90°C in 10% KOH for 10 minutes to dissolve all the tissue except cuticle. The cuticle was washed with PBS and stored in 50% glycerol. For mounting, abdomen of the fly was separated from the whole body by cutting between thorax and abdominal segment 1. Then the abdomen was cut from dorsal mid line to open the cuticle and remove all other tissues. Finally, cuticles were mounted with dorsal side facing up in 50% glycerol for imaging.

## Supporting Information

Figure S1 Expression pattern of Hox genes at embryonic stage. **A**) A 12 h old embryo is placed facing ventral side, anterior up and lateral on left right side, showing expression of Ubx (green) from parasegment (PS) 5 to 12 and that of Abd-A (red) from PS 7 to 12. Although their global expression pattern is mostly exclusive even when they are expressed in the same parasegments, they overlap in several cells (shown on the right side with arrow heads). **B**) Expression of Abd-B is seen from PS 10 to 14 and it overlaps with Abd-A in PS 10 to 12. This overlap of Abd-A and Abd-B is not only in the segments but also in the same cell (shown on the right side with arrow heads).
(TIFF)

Figure S2 Expression pattern of *71B*-Gal4. The expression of *71B*-Gal4 is shown in 32 h old pupae where HNCs (white arrowheads) have eliminated most of the LECs (yellow arrowheads) from dorsal mid line. At this stage expression of *71B*-Gal4 is seen in all the LECs but only in few HNCs stochastically. Only two segments of the early pupal abdominal epithelium is shown placed facing dorsal side, anterior side is up and lateral sides on left and right.
(TIFF)

Figure S3 Expression pattern of *Pnr*-Gal4. Upper panel shows LECs and HNCs in three segments of 26 h old pupal epithelia expressing GFP under *Pnr*-Gal4. Pupa is placed dorsal left and lateral right side. *Pnr*-Gal4 expresses in all the LECs (right) but only subset of HNCs. It expresses only in leading HNCs of the epithelia which are moving towards dorsal mid line, while HNCs at lateral side do not express *Pnr*-Gal4. Lower panel shows expression of *Pnr*-Gal4 only in leading HNCs and LECs in a 32 h old pupal epithelia. Only the abdominal region of the pupae is placed, facing dorsal side and anterior on top.
(TIFF)

Figure S4 Ventral view of wild type and *esg*-Gal4 with *abd-A RNAi* pharates. As indicated by arrows, bristles on the ventral sternite are absent in the *abd-A RNAi* context, suggesting that *abd-A* is required in HNCs for the formation of ventral epithelia.
(TIFF)

Figure S5 Expression pattern of Ubx in *esg*-Gal4 driven knockdown of *abd-A*. On knocking down of *abd-A* in HNCs using *esg*-Gal4 leads to derepression of *Ubx* in HNCs of posterior segments, while the expression of Ubx is seen only in A1 segment in the wild type (Figure 1B).
(TIFF)

Figure S6 RNAi of *Abd-B* using *71B*Gal4 and *Pnr-*Gal4. Cuticle preparations of adult abdomen is placed anterior up and dorsal right. **A**) It shows three abdominal segments (A4–6) of wild type male fly **B**) The RNAi knockdown of *Abd-B* using *71B-*Gal4 shows anteriorization of the posterior segments. The A6 sternite has bristles (red arrow) indicating partial transformation of A6 into A5 and a small extra segment (yellow arrow) suggest A7 to A6 transformation. **C**) Knockdown of *Abd-B* using *Pnr*-Gal4 show loss of pigmentation in parts of tergites closer to dorsal mid line in A4 and A5 (red arrows) suggesting partial transformation into anterior segment. These flies also showed an extra A7 segment (yellow arrow) suggesting A7 to A6 transformation.
(TIFF)

Figure S7 Over expression phenotype of *abd-A* using *Abd-B*-Gal4 driver. Over expression of *abd-A* using *Abd-B* Gal4*^119^* shows a broader A7 in female (dotted red lines) as compared to *Abd-B* Gal4^119^ alone. Both the pictures are shown at same magnification for comparison.
(TIFF)
