# Supplementary figures and images for: Role of abd-A and Abd-B in Development of Abdominal Epithelia Breaks Posterior Prevalence Rule
Source: PLoS Genet. 2014 Oct 23;10(10):e1004717. doi: 10.1371/journal.pgen.1004717 (PMC4207640; doi:10.1371/journal.pgen.1004717)

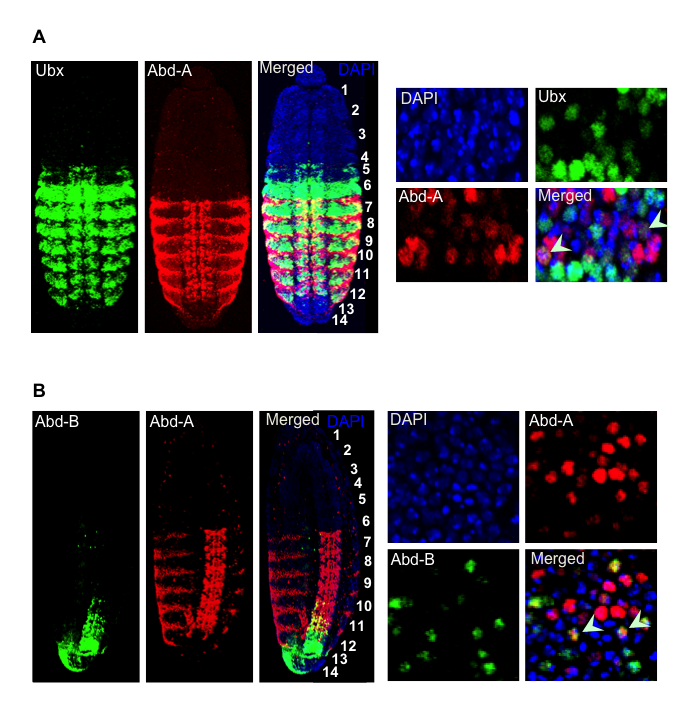

Supplement: Figure S1 — Expression pattern of Hox genes at embryonic stage. A) A 12 h old embryo is placed facing ventral side, anterior up and lateral on left right side, showing expression of Ubx (green) from parasegment (PS) 5 to 12 and that of Abd-A (red) from PS 7 to 12. Although their global expression pattern is mostly exclusive even when they are expressed in the same parasegments, they overlap in several cells (shown on the right side with arrow heads). B) Expression of Abd-B is seen from PS 10 to 14 and it overlaps with Abd-A in PS 10 to 12. This overlap of Abd-A and Abd-B is not only in the segments but also in the same cell (shown on the right side with arrow heads). (TIFF) [file pgen.1004717.s001.tiff]

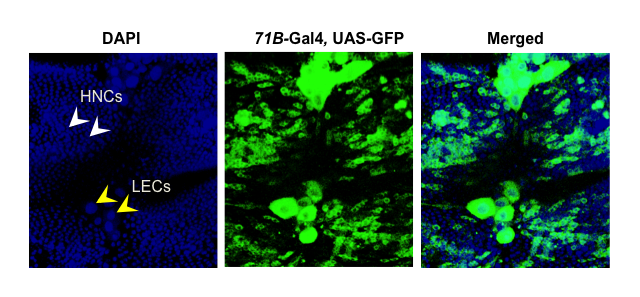

Supplement: Figure S2 — Expression pattern of 71B-Gal4. The expression of 71B-Gal4 is shown in 32 h old pupae where HNCs (white arrowheads) have eliminated most of the LECs (yellow arrowheads) from dorsal mid line. At this stage expression of 71B-Gal4 is seen in all the LECs but only in few HNCs stochastically. Only two segments of the early pupal abdominal epithelium is shown placed facing dorsal side, anterior side is up and lateral sides on left and right. (TIFF) [file pgen.1004717.s002.tiff]

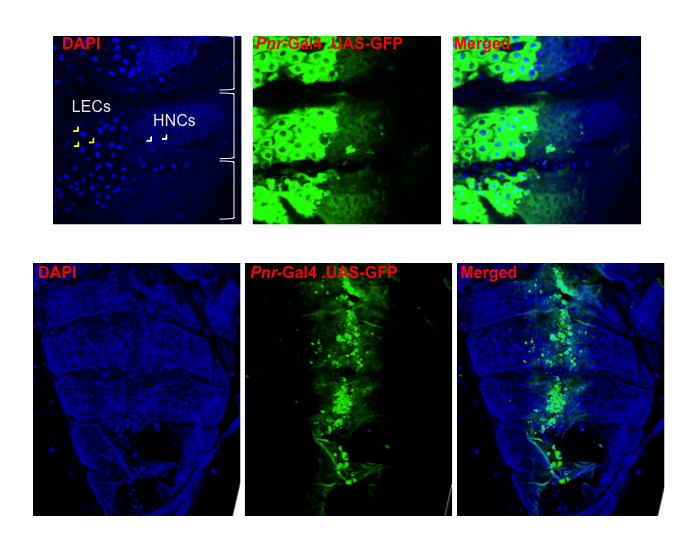

Supplement: Figure S3 — Expression pattern of Pnr-Gal4. Upper panel shows LECs and HNCs in three segments of 26 h old pupal epithelia expressing GFP under Pnr-Gal4. Pupa is placed dorsal left and lateral right side. Pnr-Gal4 expresses in all the LECs (right) but only subset of HNCs. It expresses only in leading HNCs of the epithelia which are moving towards dorsal mid line, while HNCs at lateral side do not express Pnr-Gal4. Lower panel shows expression of Pnr-Gal4 only in leading HNCs and LECs in a 32 h old pupal epithelia. Only the abdominal region of the pupae is placed, facing dorsal side and anterior on top. (TIFF) [file pgen.1004717.s003.tiff]

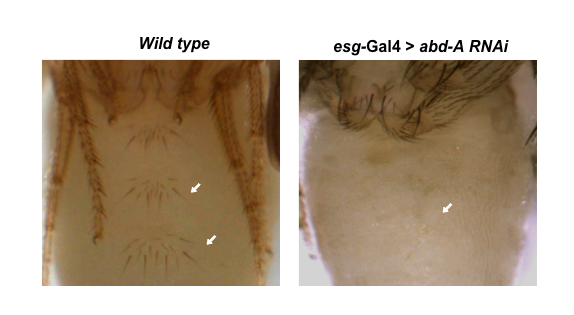

Supplement: Figure S4 — Ventral view of wild type and esg-Gal4 with abd-A RNAi pharates. As indicated by arrows, bristles on the ventral sternite are absent in the abd-A RNAi context, suggesting that abd-A is required in HNCs for the formation of ventral epithelia. (TIFF) [file pgen.1004717.s004.tiff]

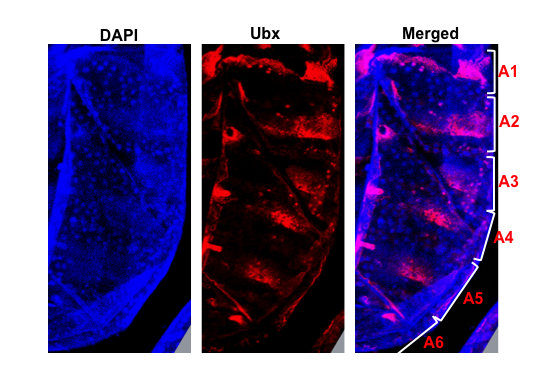

Supplement: Figure S5 — Expression pattern of Ubx in esg-Gal4 driven knockdown of abd-A. On knocking down of abd-A in HNCs using esg-Gal4 leads to derepression of Ubx in HNCs of posterior segments, while the expression of Ubx is seen only in A1 segment in the wild type (Figure 1B). (TIFF) [file pgen.1004717.s005.tiff]

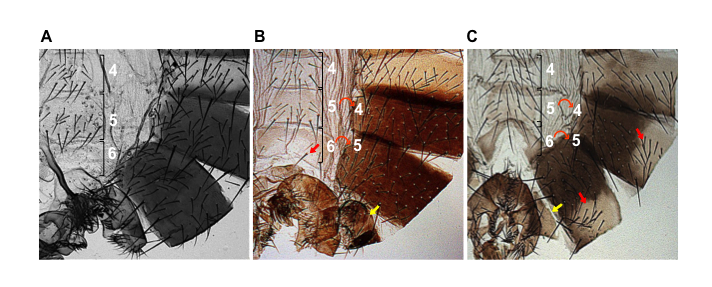

Supplement: Figure S6 — RNAi of Abd-B using 71BGal4 and Pnr-Gal4. Cuticle preparations of adult abdomen is placed anterior up and dorsal right. A) It shows three abdominal segments (A4–6) of wild type male fly B) The RNAi knockdown of Abd-B using 71B-Gal4 shows anteriorization of the posterior segments. The A6 sternite has bristles (red arrow) indicating partial transformation of A6 into A5 and a small extra segment (yellow arrow) suggest A7 to A6 transformation. C) Knockdown of Abd-B using Pnr-Gal4 show loss of pigmentation in parts of tergites closer to dorsal mid line in A4 and A5 (red arrows) suggesting partial transformation into anterior segment. These flies also showed an extra A7 segment (yellow arrow) suggesting A7 to A6 transformation. (TIFF) [file pgen.1004717.s006.tiff]

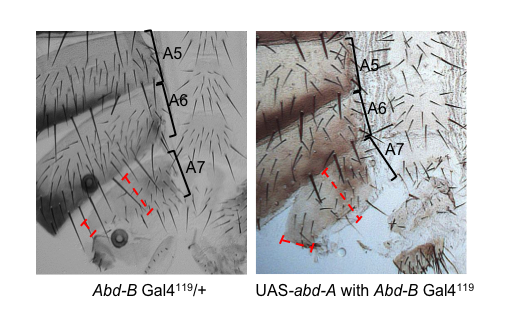

Supplement: Figure S7 — Over expression phenotype of abd-A using Abd-B-Gal4 driver. Over expression of abd-A using Abd-B Gal4119 shows a broader A7 in female (dotted red lines) as compared to Abd-B Gal4119 alone. Both the pictures are shown at same magnification for comparison. (TIFF) [file pgen.1004717.s007.tiff]
